# Comprehensive metabolome analyses reveal *N*-acetylcysteine-responsive accumulation of kynurenine in systemic lupus erythematosus: implications for activation of the mechanistic target of rapamycin

**DOI:** 10.1007/s11306-015-0772-0

**Published:** 2015-01-20

**Authors:** Andras Perl, Robert Hanczko, Zhi-Wei Lai, Zachary Oaks, Ryan Kelly, Rebecca Borsuk, John M. Asara, Paul E. Phillips

**Affiliations:** 1 Division of Rheumatology, Department of Medicine, College of Medicine, Upstate Medical University, State University of New York, Syracuse, NY 13210 USA; 2 Department of Microbiology and Immunology, College of Medicine, Upstate Medical University, State University of New York, 750 East Adams Street, Syracuse, NY 13210 USA; 3 Department of Biochemistry and Molecular Biology, College of Medicine, Upstate Medical University, State University of New York, Syracuse, NY 13210 USA; 4 Division of Signal Transduction, Department of Medicine, Beth Israel Deaconess Medical Center, Harvard Medical School, Boston, MA USA

**Keywords:** Pentose phosphate pathway, Oxidative stress, Kynurenine, mTOR, *N*-acetylcysteine

## Abstract

Systemic lupus erythematosus (SLE) patients exhibit depletion of the intracellular antioxidant glutathione and downstream activation of the metabolic sensor, mechanistic target of rapamycin (mTOR). Since reversal of glutathione depletion by the amino acid precursor, *N*-acetylcysteine (NAC), is therapeutic in SLE, its mechanism of impact on the metabolome was examined within the context of a double-blind placebo-controlled trial. Quantitative metabolome profiling of peripheral blood lymphocytes (PBL) was performed in 36 SLE patients and 42 healthy controls matched for age, gender, and ethnicity of patients using mass spectrometry that covers all major metabolic pathways. mTOR activity was assessed by western blot and flow cytometry. Metabolome changes in lupus PBL affected 27 of 80 KEGG pathways at FDR p < 0.05 with most prominent impact on the pentose phosphate pathway (PPP). While cysteine was depleted, cystine, kynurenine, cytosine, and dCTP were the most increased metabolites. Area under the receiver operating characteristic curve (AUC) logistic regression approach identified kynurenine (AUC = 0.859), dCTP (AUC = 0.762), and methionine sulfoxide (AUC = 0.708), as top predictors of SLE. Kynurenine was the top predictor of NAC effect in SLE (AUC = 0.851). NAC treatment significantly reduced kynurenine levels relative to placebo in vivo (raw p = 2.8 × 10^−7^, FDR corrected p = 6.6 × 10^−5^). Kynurenine stimulated mTOR activity in healthy control PBL in vitro. Metabolome changes in lupus PBL reveal a dominant impact on the PPP that reflect greater demand for nucleotides and oxidative stress. The PPP-connected and NAC-responsive accumulation of kynurenine and its stimulation of mTOR are identified as novel metabolic checkpoints in lupus pathogenesis.

**Electronic supplementary material:**

The online version of this article (doi:10.1007/s11306-015-0772-0) contains supplementary material, which is available to authorized users.

## Introduction

Systemic lupus erythematosus (SLE) is a potentially fatal autoimmune inflammatory disease of unknown etiology. Dysfunction of T and B cells drives anti-nuclear antibody production through the release of oxidized immunogenic nuclear materials from necrotic rather than apoptotic cells (Perl 2013). Importantly, lupus T cells exhibit mitochondrial dysfunction, which is characterized by elevated mitochondrial transmembrane potential (Δψ_m_) or persistent mitochondrial hyperpolarization (MHP) that mediates diminished activation-induced apoptosis and predisposes T cells to pro-inflammatory death via necrosis (Gergely et al. 2002; Lai et al. 2013). MHP is associated with increased O_2_ consumption and electron transport chain activity (Doherty et al. 2014), promoting the transfer of electrons to O_2_, thus resulting in the enhanced production of reactive oxygen intermediates (ROI), i.e. oxidative stress (Gergely et al. 2002), and the depletion of reduced glutathione (GSH) (Gergely et al. 2002). Both GSH depletion (Shah et al. 2011) and oxidative stress have been confirmed and implicated in T cells dysfunction in SLE (Li et al. 2014).

In turn, the mechanistic/mammalian target of rapamycin (mTOR) is a sensor of oxidative stress and regulator of T cell lineage specification (Chi 2012). As recently documented, *N*-acetylcysteine (NAC), a precursor of GSH and antioxidant in itself, reversed the depletion of GSH, and it also blocked mTOR activation and improved disease activity in SLE patients (Lai et al. 2012). However, NAC failed to moderate MHP and ROI production, suggesting that oxidative stress may not directly activate mTOR. Although mTOR is widely recognized as a global sensor and integrator of nutrient pathways (Kim et al. 2002), in particular amino acids, the individual metabolites have not been identified (Jewell et al. 2013). Here, we document global changes in the metabolome of peripheral blood lymphocytes (PBL) from SLE patients in comparison to healthy controls matched at each blood donation for patients’ age within 10 years, gender, and ethnicity. Metabolome changes in lupus PBL were most prominently impacted by the pentose phosphate pathway (PPP). Oxidative stress was evidenced by the depletion of cysteine and the increased levels of cystine and methionine sulfoxide (Met-SO). Area under the receiver operating characteristic (ROC) curve (AUC) logistic regression approach identified the amino acid kynurenine (Kyn) to have the greatest specificity and sensitivity for distinguishing the metabolomes of lupus and control PBL. NAC treatment reduced Kyn, while it enhanced NADPH levels in PBL of SLE patients in vivo. Moreover, Kyn induced mTOR activation in T cells in vitro. This study thus identifies Kyn accumulation as a potential contributor to mTOR activation and metabolic target of the therapeutic action by NAC in patients with SLE.

## Methods

### Human subjects

36 SLE patients enrolled in double-blind placebo-controlled treatment trial with *N*-acetylcysteine (NAC) were investigated. This study has been approved by the Food and Drug Administration (IND No: 101,320; clinicaltrials.gov identifier: NCT00775476). The clinical trial design, eligibility criteria, randomization, blinding, monitoring of safety, tolerance and efficacy on NAC in patients with SLE was recently documented (Lai et al. 2012). SLE disease activity was assessed using the British Isles Lupus Assessment Group (BILAG) (Isenberg et al. 2005) and systemic lupus erythematosus disease activity index (SLEDAI) (Hochberg 1997). Fatigue was estimated using the fatigue assessment scale (FAS) (Michielsen et al. 2003).

The mean (±SEM) age of patients was 44.6 (±1.8) years, ranging between 25 and 64 years, as earlier described (Lai et al. 2012). 34 patients were females including 30 Caucasians, two African-Americans, and two Hispanic. 2 patients were Caucasian males. 42 healthy subjects were individually matched for each patient blood donation for age within ten years, gender, and ethnic background and freshly isolated cells were studied in parallel as controls for immunological studies. The mean (±SEM) age of controls was 44.4 (±1.7) years, ranging between 22 and 63 years. 39 controls were females including 36 Caucasians, two African-Americans, and one Hispanic. 3 controls were Caucasian males. SLE patients were randomized to receive either placebo or NAC in one of three treatment arms of increasing doses: 600, 1,200, or 2,400 mg twice daily for 3 months. 12 patients were enrolled per dosing group, 9 received NAC while 3 received placebo. For each patient visit, we obtained blood from healthy donors matched for age (within one decade), gender, and ethnicity, to be used as a control for flow cytometry measurement of mitochondrial function, gene expression, and metabolite analysis. Each patient provided five blood samples for metabolomic studies (visit 1/pretreatment, visit 2/after 1 month treatment, visit 3/after 2 months treatment, visit 4/after 3 months treatment, visit 5/after 1 month washout). 42 healthy controls have also donated blood to use as control for flow cytometry of live cells as well as for the metabolomic, gene expression and signaling studies.

### Metabolyte measurements by LC–MS/MS

5 × 10^6^ monocyte-depleted peripheral blood lymphocytes (PBL) were washed in PBS, resuspended in 100 µl of 80 % methanol (−80 °C). After freezing at −80 °C and thawing once, the sample was centrifuged at 13,000 x g for 30 min at 4 °C, and 100 µl of supernatant was saved. A 2nd 100 µl of 80 % methanol (−80 °C) was added to the pellet, the sample was vortexed, centrifuged at 13,000×*g* for 30 min at 4 °C, and the 2nd 100 µl of supernatant was saved. The two 100-µl supernatants were combined, dried in a SpeedVac (Savant AS160, Farmingdale, NY), and stored −80 °C until analysis. Each sample was resuspended in 20 μl of LC/MS grade water and 10 μl per sample was injected into a 5500 QTRAP, a hybrid triple quadrupole/linear ion trap mass spectrometer, using a quantitative polar metabolomics profiling platform with selected reaction monitoring (SRM) that covers all major metabolic pathways. The platform uses hydrophilic interaction liquid chromatography with positive/negative ion switching to analyze 258 metabolites (289 Q1/Q3 transitions) from a single 15-min targeted liquid chromatography–tandem mass spectrometry (LC–MS/MS) acquisition with a 3-ms dwell time and a 1.55-s duty cycle time (Yuan et al. 2012).

A healthy subject matched for age within 10 years, gender, and ethnicity was recruited upon each patient visit. The blood samples of patients and matched controls were processed in parallel on ice, stored in parallel at −80 °C until injected in the same run for LC–MS/MS analysis.

### Metabolic pathway and statistical analyses

Quantitative enrichment analysis of 258 detected metabolites was utilized for pathway analysis employing the web-based MetaboAnalyst 2.0 software (Xia and Wishart 2011). Upon each patient visit, a healthy subject matched for age within 10 years, gender, and ethnicity was recruited. The blood samples were processed in parallel. The patients and matched healthy subjects were injected in the same run. The signal stability was assured by normalizing the controls between runs to the sum of all signals between separate runs using Metaboanalyst (Xia and Wishart 2011). The enrichment analysis was based on global analysis of covariance (Ancova). A Google-map style interactive visualization system was utilized for data exploration and creation of a 3-level graphical output: metabolome view, pathway view, and compound view. The ‘metabolome view’ shows all metabolic pathways arranged according to the scores from enrichment analysis (*y* axis: −log p) and from topology analysis (*x* axis: impact: number of detected metabolites with significant p value) (Xia and Wishart 2011). The matched metabolites are highlighted according to their Holm p values. The Holm p is the p value adjusted by Holm-Bonferroni method (Holm 1979). The pathway topology analysis used two well-established node centrality measures to estimate node importance: degree centrality and betweeness centrality. Degree centrality depends on the number of links connected to a given node. For directed pathway graphs, there are two types of degrees: in-degree for links came from other nodes, and out-degree for links initiated from the current node. Here, we only considered the out-degree for node importance measure. Upstream nodes are considered to have regulatory roles for the downstream nodes, and not vice versa. The betweeness centrality measures the number of shortest paths going through the node. Since metabolic networks are directed, we use relative-betweeness centrality for a metabolite importance measure (Tuikkala et al. 2012). The degree centrality measures focus more on local connectivities, while the betweeness centrality measures focus more on global network topology. The node importance values calculated from centrality measures were further normalized by the sum of the importance of the pathway. Therefore, the total/maximum importance of each pathway reflects the importance measure of each metabolite node that is actually the percentage relative to the total pathway importance, and the pathway impact value is the cumulative percentage from the matched metabolite nodes. The altered compounds have been grouped and presented together for each pathway.

The impact of NAC on metabolic changes relative to placebo was investigated by performing a two-factor (NAC versus placebo) time series analysis (changes relative to baseline/visit 1) within individual subjects. Two-way within-subject ANOVA was performed, and the interaction of drug with time was analyzed by comparing data acquired after treatment for 1 month (visit 2), 2 months (visit 3), and 3 months (visit 4) relative to baseline (visit 1). Using MetaboAnalyst, we also performed ANOVA-simultaneous component analysis (ASCA), which is a multivariate extension of ANOVA. It is designed to identify the major patterns associated with each factor. This implementation supports the ASCA model for two factors with one interaction effect. The algorithm first partitions the overall data variance (X) into individual variances induced by each factor (A and B), as well as by the interactions (AB). The formula is shown below with (E) indicates the residual Errors: X = A + B + AB + E. The SCA part applies principal component analysis (PCA) to A, B, AB to summarize major variations in each partition. The significant variables are identified based on the leverage and the squared prediction errors (SPE) associated with each variables. Variables with low SPE and higher leverage are modeled well after the major patterns. Pre-treatment samples obtained at visit 1 were also compared to samples obtained at visits 2–4 during biologically and clinical effective administration of NAC at doses of 2.4 and 4.8 g/day by ANCOVA.

Metabolite concentrations were evaluated for their ability to discriminate between SLE and control subjects by partial least squares-discriminant analysis (PLS-DA) using MetaboAnalyst (Xia and Wishart 2011). PLS is a supervised method that uses a multi-variate regression technique to extract via linear combination of metabolites (X) the information that can predict the subject group membership (Y). The classification and cross validation were performed using the wrapper function offered by the caret package in MetaboAnalyst software (Xia and Wishart 2011). In order to assess that the class discrimination is statistically significant, a permutation test was performed. In each permutation, a PLS-DA model was built between the data (X) and the permuted class labels (Y) using the optimal number of components determined by cross validation for the model based on the original class assignment. The ratio of the between sum of the squares and the within sum of squares (B/W-ratio) for the class assignment prediction of each model was calculated. PLS-DA models were validated by permutation test p value <0.05. Contribution of individual metabolites to PLS-DA was assessed by variable importance in projection (VIP) and coefficient scores.

Individual compounds were also compared between control and lupus PBL by paired or unpaired t-test with Welch’s correction using Prism software (GraphPad, San Diego, CA). Pearson’s correlations of normalized metabolite concentrations with disease activity and flow cytometry parameters were determined with MetaboAnalyst and Prism. Areas under the receiver operating characteristic (ROC) curve (AUC) logistic regression approach was used for evaluating the specificity and sensitivity of individual metabolites and metabolite ratios for distinguishing lupus and control PBL as well as NAC-treated and untreated lupus PBL.

### Western blot analyses

Whole cell protein lysates were prepared by lysis in radio-immunoprecipitation assay buffer (150 mM NaCl, 2 % NP-40, 0.5 % sodium deoxycholate, 0.1 % SDS, 50 mM Tris pH 8.0, 1 mM PMSF, 1 μg/ml aproptinin, 1 μg/ml pepstatin, 1 μg/ml leupeptin, 1 mM NaF, 1 mM sodium orthovanadate, 0.1 mM sodium molybdate, 10 mM sodium pyrophosphate) at a density of 4 × 10^7^ cells/ml on ice, followed by addition of equal volumes of Laemmli protein sample buffer (60 mM Tris–Cl pH 6.8, 2 % SDS, 10 % glycerol, 5 % β-mercaptoethanol, 0.01 % bromophenol blue) and heated to 95 °C for 5 min prior to separation on SDS-PAGE gels and transfer to 0.45 μm nitrocellulose membranes. 4E-BP1 (4E-BP1; Cat no 9644) and phospho-4E-BP1 (p4E-BP1 Thr 37/46, Cat no 2855) antibodies were obtained from Cell Signaling. p70 S6 kinase (S6 K, Cat no sc-8418) and phospho-p70 S6 kinase (pS6K Thr 389, Cat no sc-8416) were from Santa Cruz Biotechnology. Reactivity to primary antibodies was detected with horseradish peroxidase-conjugated secondary antibodies (Jackson, West Grove, PA) and visualized by enhanced chemiluminescence (Western Lightning Chemiluminescence Reagent Plus, GE Health Care/PerkinElmer Life Sciences, Inc., Boston, Massachusetts). Automated densitometry was used to quantify the levels of mTOR substrate protein expression relative to β-actin using a Kodak Image Station 440CF with Kodak 1D Image Analysis Software (Eastman Kodak Company, Rochester, NY).

### Flow cytometry detection of mTOR activity

PBL from eight healthy subjects were incubated with kynurenine (Kyn, 0.05, 0.1, 0.5, and 1), l-homocysteic acid (HCA, 100, 500 μM, 1 mM, 5 mM) and dibutyryl cAMP (db-cAMP, 500 μM) for 24 h. Tryptophan was used as a control amino acid (100 μM, 1 mM, 10 mM). All chemicals were obtained from Sigma (St. Louis, MO).The effect of these metabolites on mTOR activity was measured via phosphorylation S6RP (Lai et al. 2012). T-cell subsets were analyzed by staining with antibodies to CD4, CD8, and CD25. For detection of mTOR activity and FoxP3 expression, cells were permeabilized with Cytofix/CytopermPlus (eBiosciences) and stained with AlexaFluor-488-conjugated antibody to pS6RP (Cell Signaling; Beverly, MA; Cat. No. 4851) and AlexaFluor-647-conjugated antibody to FoxP3 (BioLegend, San Diego, CA; Cat No 320014), as earlier described (Lai et al. 2012).

## Results

### Metabolome analysis reveals dominant impact of SLE on the pentose phosphate pathway, nucleotide, cysteine, and kynurenine metabolism

**Fig. 1:**
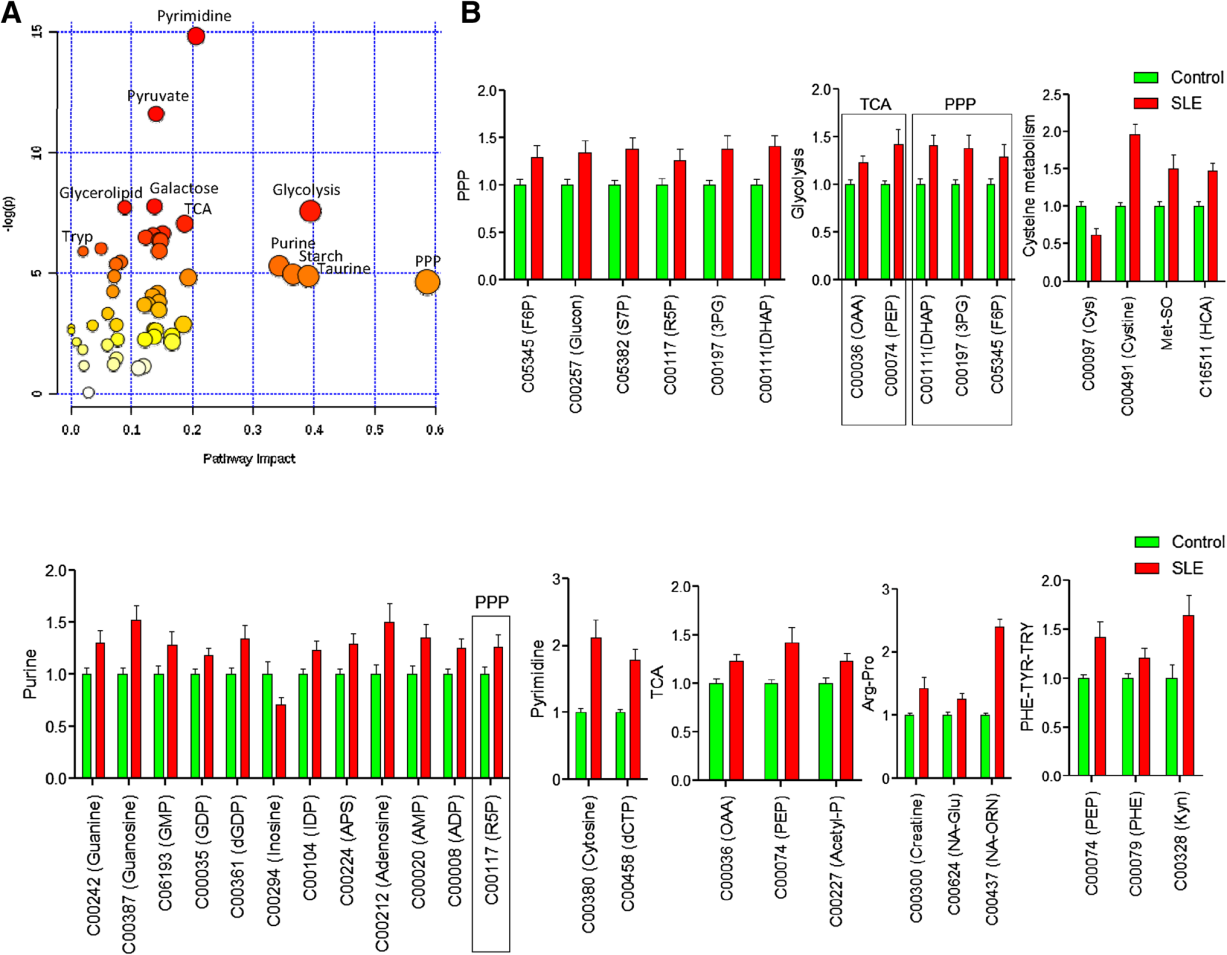
Metabolic pathway analysis of quantitative changes in compound concentrations in PBL samples of 36 SLE patients relative to 42 healthy subjects matched for age within 10 years, gender, and ethnic background. **a** The ‘metabolome view’ shows all 80 KEGG metabolic pathways arranged according to the scores from enrichment analysis (*y* axis: −log p) and topology analysis (*x* axis: impact: number of detected metabolites in a pathway with significant p values). **b** Metabolites with altered levels in lupus PBL relative to matched healthy control PBL normalized to 1.0 for each compound. 6 of 34 altered metabolites can enter multiple pathways: 4 of such 6 metabolites are substrates in the PPP, while 2 metabolites are common with the TCA. Only 2/34 altered metabolites were depleted, cysteine and inosine

Quantitative enrichment analysis of 258 measured metabolites revealed robust changes in the metabolome of SLE patients’ PBL relative to those of healthy controls matched for age within 10 years, gender, and ethnic background. The metabolome changes affected 27 of 80 pathways in the KEGG database at false discovery rate (FDR) p value <0.05, with the most prominent impact on the pentose phosphate pathway (PPP, Fig. 1a, Fig. S1 and Table 1). The pathway changes were driven by the accumulation of 32 among 34 metabolites altered in lupus PBL (Fig. 1b) 0.11 of these metabolites are involved in multiple pathways, with 4/11 being substrates in the PPP (Fig. 1b). After the PPP, glycolysis, starch (carbohydrate), taurine, as well as purine and pyrimidine metabolism were the most affected pathways (Table 1). Only 2 metabolites were depleted, cysteine and inosine. Cysteine is a substrate of 6 pathways affected by SLE: GSH, taurine, thiamine, Gly-Ser-Thr, Cys-Met, and sulfur metabolism (Fig. S1). Moreover, we also observed the accumulation of kynurenine (Kyn), which is a product of tryptophan catabolism (Aune and Pogue 1989) and regulator of the PPP (Fabregat et al. 1987).

**Table 1:** Metabolome effects of lupus by quantitative enrichment analysis of 209 metabolites in PBL of SLE patients in comparison to those of healthy controls matched for age within 10 years, gender, and ethnic background

	Total	Hits	Raw p	−LOG (p)	FDR p	Impact
Pentose phosphate pathway (PPP)	32	14	0.0097581	4.6297	0.022621	0.58698
Glycolysis	31	11	0.0005255	7.5511	0.005361	0.39538
Taurine and hypotaurine	20	4	0.007702	4.8663	0.019756	0.39131
Starch and sucrose	50	7	0.0070929	4.9487	0.019756	0.36587
Purine metabolism	92	20	0.0050401	5.2903	0.01512	0.34287
Pyrimidine metabolism	60	10	3.73E−07	14.8	1.90E−05	0.2062
Phenylalanine, tyrosine and tryptophan biosynthesis	27	7	0.0081346	4.8116	0.019756	0.19356
Citrate cycle (TCA cycle)	20	5	0.0008794	7.0362	0.007475	0.1875
Glyoxylate and dicarboxylate metabolism	50	5	0.0013318	6.6212	0.008382	0.15152
Cysteine and methionine metabolism	56	5	0.0018078	6.3156	0.008382	0.14815
Arginine and proline metabolism	77	7	0.0017812	6.3305	0.008382	0.14563
Nicotinate and nicotinamide metabolism	44	4	0.0027461	5.8976	0.01009	0.14545
Glutathione metabolism	38	6	0.022229	3.8064	0.043602	0.14517
Alanine, aspartate and glutamate metabolism	24	3	0.015304	4.1797	0.03252	0.14286
Pyruvate metabolism	32	6	9.36E−06	11.579	0.000239	0.14035
Galactose metabolism	41	6	0.0004329	7.745	0.005361	0.13792
Glycine, serine and threonine metabolism	48	8	0.0014171	6.5591	0.008382	0.1356
Nitrogen metabolism	39	7	0.017404	4.0511	0.035504	0.13461
Phenylalanine metabolism	45	3	0.024612	3.7045	0.045465	0.12727
Glycerophospholipid metabolism	39	4	0.0015563	6.4654	0.008382	0.12329
Pantothenate and CoA biosynthesis	27	4	0.024961	3.6904	0.045465	0.12121
Glycerolipid metabolism	32	5	0.0004521	7.7015	0.005361	0.08888
Fructose and mannose metabolism	48	4	0.0042946	5.4504	0.014602	0.08197
Sulfur metabolism	18	3	0.0047212	5.3557	0.015049	0.07407
Tryptophan metabolism	79	2	0.0077869	4.8553	0.019756	0.07142
Thiamine metabolism	24	2	0.01435	4.244	0.031819	0.06896
Pentose and glucuronate interconversions	53	5	0.0024615	6.007	0.01009	0.05001

The metabolome changes affected 27 of 80 pathways in the KEGG database at false discovery rate (FDR) p value <0.05. The pathways are arranged by the relative impact of the number of metabolites affected and their node of importance (Xia et al. 2012)

**Fig. 2:**
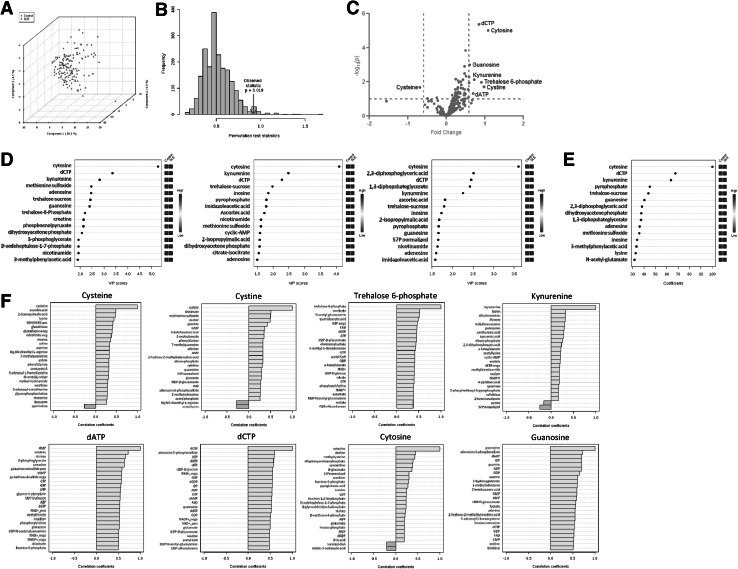
Partial least squares-discriminant analysis (PLS-DA) of metabolite concentrations in lupus and control PBL. Samples obtained from 36 lupus patients before initiation of treatment with NAC, e.g. Visit 1, were compared to samples from 42 healthy subjects processed in parallel. **a** 3-dimensional score plot of PLS-DA using components 1, 2, and 3, accounting for 20.2, 6.9, and 4.5 % of the total variance. **b** Validation of PLS-DA by permutation test p value <0.001. **c** Volcano plot is a combination of fold change (FC, log_2_ FC: X axis) and *t* test p values (−log10 p: Y axis). **d** Variable importance in projection (VIP) scores of 15 top contributors to PLS-DA components 1–3. **e** Coefficient-based importance measures of top 15 contributors to components 1–3. **f** Correlation plot showing the compounds as *horizontal bars*, with colors in *light pink* indicating positive correlations and those in *light blue* indicating negative correlations with top lupus-associated metabolites identified in the volcano plot (**c**). Values reflect Pearson’s correlation coefficients between metabolite concentrations at FDR p < 0.05 calculated by MetaboAnalyst (Color figure online)

Partial least squares-discriminant analysis (PLS-DA) of metabolite concentrations discriminated between lupus and control PBL (Fig. 2a), as validated by permutation test p value < 0.001 (Fig. 2b).Three component matrices accounted for 20.2, 6.9, and 4.5 % of the total variance (Fig. 2a). Top contributors to the PLS-DA included nucleotides and their amino acid and PPP sugar precursors. Individually, cytosine, dCTP, guanosine, cystine, and Kyn were the most increased while cysteine was the most depleted metabolite in lupus PBL (Fig. 2c).These metabolites were the greatest contributors to PLS-DA components 1–3 based on both variable importance in projection (VIP) (Fig. 2d) and coefficient scores (Fig. 2e). The pyrimidine nucleotide cytosine, dCTP, and Kyn had the top VIP and coefficient scores (Fig. 2d, e). Although methanol extraction, which was utilized to process cell extracts for LC–MS/MS studies, was not sensitive for detection of GSH, oxidative stress was evidenced by the depletion of cysteine and the accumulation of cystine and Met-SO (Fig. 1b, c). Correlation analysis with metabolites exhibiting dominant changes between lupus and control PBL (Fig. 2c) unveiled additional trends of oxidative stress, such as positive correlations of cysteine with ascorbate, GSH, and GSH/GSSG ratio (Fig. 2f).

**Fig. 3:**
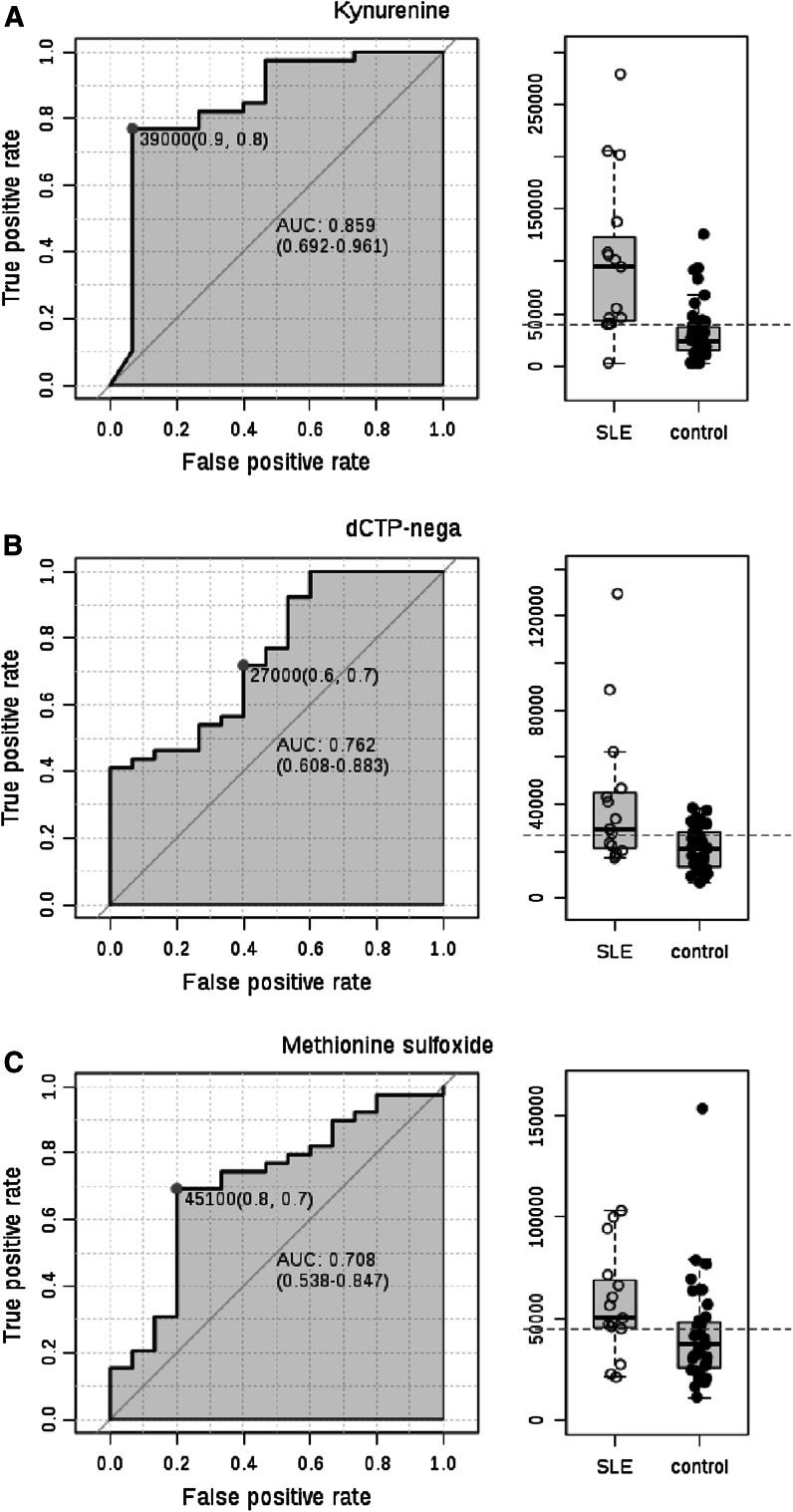
Discrimination of the metabolome of lupus and control PBL based on area under the receiver operating characteristic (ROC) curve (AUC) logistic regression approach. For each of the top three discriminating metabolites, the *left panel* shows the AUC confidence interval, true positive and false positive rates, and confidence interval (CI), the *right panel* shows the concentrations of metabolites in PBL before (baseline) and during NAC treatment (NAC). AUC logistic regression approach identified Kyn (AUC = 0.859), dCTP (AUC = 0.762), and Met-SO (AUC = 0.708) to have the greatest specificity and sensitivity for distinguishing the metabolome of lupus and control PBL

Area under the receiver operating characteristic (ROC) curve (AUC) logistic regression approach identified Kyn (AUC = 0.859), dCTP (AUC = 0.762), and Met-SO (AUC = 0.708) to have the greatest specificity and sensitivity for distinguishing the metabolomes of lupus and control PBL (Fig. 3).

### Correlation of metabolite concentrations with disease activity

Concentrations of 18, 16, and 32 compounds showed significant correlation at FDR p < 0.05 with SLEDAI (Table 2), BILAG (Table 3), and FAS disease activity scores in 36 SLE patients unexposed to NAC (Table 4), respectively. 9 compounds showed correlation with all three disease activity scores: dihydroxy-acetone-phosphate (DHAP), oxaloacetate (OAA), glucose 1-phosphate (G1P), hexose-phosphate, deoxyribose-phosphate, uracil, acetoacetate, geranyl pyrophosphate (GPP), and indole-3-carboxylic acid. DHAP connects the PPP while OAA connects the TCA with glycolysis. Glucose-1-phosphate is generated during glycogenolysis. It can be converted into G6P for metabolism through the PPP or glycolysis (Mayes 1993). Hexose-phosphate and deoxyribose-phosphate represent structural isomers of compounds that are involved in the PPP, glycolysis, and nucleotide metabolism. Acetoacetate is the product of amino acid and fatty acid catabolism. GPP is an intermediate towards the biosynthesis of farnesyl pyrophosphate, geranylgeranyl pyrophosphate, and cholesterol (Holstein and Hohl 2004). Rab GTPases require geranylgeranyl pyrophosphate to attach to endosomes and to regulate endosome traffic, which is increased in lupus T cells (Fernandez et al. 2009).

**Table 2:** Correlation of metabolite concentrations with SLEDAI in PBL of 36 lupus patients that have not been exposed to NAC

	Correlation	t-stat	p value	FDR
SLEDAI	1	Inf	0	0
BILAG	0.77074	8.8892	3.72E−12	4.41E−10
*DHAP*	0.53154	4.6114	2.49E−05	0.00197
*Hexose-phosphate*	0.49239	4.1572	0.000116	0.005688
Betaine	0.49144	4.1466	0.00012	0.005688
*Oxaloacetate*	0.48506	4.076	0.000151	0.00598
*Deoxyribose-phosphate*	0.47252	3.9399	0.000236	0.007987
*G1P*	0.46369	3.8459	0.000319	0.009455
2-oxobutanoate	0.45647	3.7701	0.000406	0.010696
*Acetoacetate*	0.4289	3.4889	0.000973	0.023066
*Acetylphosphate*	0.41531	3.3549	0.001458	0.031419
P-hydroxybenzoate	0.40933	3.2968	0.001733	0.034228
1,3-diphopshateglycerate	0.4024	3.2301	0.002109	0.038445
*Uracil*	0.39868	3.1945	0.002339	0.03907
UDP-*N*-acetyl-glucosamine	−0.39667	−3.1754	0.002473	0.03907
Spermine	0.38916	3.1045	0.003033	0.044928
*GPP*	0.38612	3.076	0.00329	0.045871
7-methylguanosine	−0.38033	−3.0219	0.003835	0.048726
Xanthine	0.37934	3.0128	0.003935	0.048726
*Indole-3-carboxylic acid*	0.37765	2.9971	0.004112	0.048726

Compounds in italics correlated at FDR p < 0.05 with SLE, BILAG, and FAS scores

**Table 3:** Correlation of metabolite concentrations with BILAG in PBL of 36 lupus patients that have not been exposed to NAC

	Correlation	t-stat	p value	FDR
BILAG	1	Inf	0	0
SLEDAI	0.77074	8.8892	3.72E−12	4.41E−10
*Acetylphosphate*	0.52377	4.5183	3.43E−05	0.002459
*Deoxyribose-phosphate*	0.51909	4.4628	4.15E−05	0.002459
*Hexose-phosphate*	0.5006	4.2495	8.52E−05	0.004041
NADP	0.48776	4.1058	0.000137	0.005423
*GPP*	0.47847	4.0041	0.000192	0.005973
*Uracil*	0.47702	3.9884	0.000202	0.005973
*G1P*	0.46738	3.8849	0.000282	0.00722
*DHAP*	0.46433	3.8527	0.000312	0.00722
Flavone	0.46118	3.8194	0.000347	0.00722
Pyrophosphate	0.45965	3.8033	0.000366	0.00722
*Indole-3-carboxylic acid*	0.4546	3.7505	0.000432	0.007877
*Acetoacetate*	0.40659	3.2703	0.001874	0.029561
Glycolate	0.40395	3.245	0.002019	0.029561
Asparagine	−0.40296	−3.2354	0.002076	0.029561
*Oxaloacetate*	0.4022	3.2282	0.00212	0.029561
dATP	−0.39857	−3.1935	0.002346	0.03089

Compounds in italics correlated at FDR p < 0.05 with SLE, BILAG, and FAS scores

**Table 4:** Correlation of metabolite concentrations with FAS fatigue scores in PBL of 36 lupus patients that have not been exposed to NAC

	Correlation	t-stat	p value	FDR
FAS	1	3.49E+08	0	0
SLEDAI	0.69858	7.1744	2.15E−09	2.56E−07
BILAG	0.61666	5.7563	4.18E−07	3.33E−05
*Dihydroxy-acetone-phosphate*	0.59713	5.4703	1.19E−06	7.09E−05
*Oxaloacetate*	0.57832	5.2093	3.04E−06	0.000145
*Acetoacetate*	0.57098	5.1108	4.32E−06	0.000172
Flavone	0.56337	5.0108	6.17E−06	0.000211
2,3-Diphosphoglyceric acid	0.53154	4.6113	2.49E−05	0.000745
*Indole-3-carboxylic acid*	0.52839	4.5735	2.84E−05	0.000755
Quinolinate	0.52241	4.5021	3.63E−05	0.000853
1,3-diphopshateglycerate	0.52046	4.4791	3.93E−05	0.000853
Phosphoenolpyruvate	0.50927	4.3485	6.11E−05	0.001218
*Deoxyribose-phosphate*	0.50568	4.3073	7.02E−05	0.001291
UDP-N-acetyl-glucosamine	−0.48004	−4.0211	0.000181	0.003093
*Uracil*	0.47805	3.9995	0.000194	0.003098
Acetylcarnitine DL	0.4659	3.8692	0.000296	0.004424
Betaine	0.45695	3.7751	0.0004	0.00562
*Glucose-1-phosphate*	0.45371	3.7413	0.000445	0.005907
Pyrophosphate	0.44728	3.6749	0.000548	0.006894
Citraconic acid	0.44003	3.6009	0.00069	0.008244
*Gpp*	0.43595	3.5597	0.000784	0.008919
Methionine	−0.43294	−3.5293	0.00086	0.009345
Damp	−0.41094	−3.3124	0.001655	0.017196
Kynurenic acid	0.40727	3.2769	0.001838	0.018304
Glucosamine	0.40158	3.2223	0.002157	0.020269
2-oxobutanoate	0.4008	3.2148	0.002205	0.020269
Hydroxyphenylacetic acid	0.39148	3.1263	0.002849	0.025185
P-hydroxybenzoate	0.39019	3.1141	0.002951	0.025185
*Hexose-phosphate*	0.38844	3.0977	0.003092	0.025486
Asparagine	−0.37362	−2.9599	0.004563	0.036352
Shikimate 3-phosphate	0.36996	2.9263	0.005009	0.038619
Nicotinate	0.36814	2.9096	0.005246	0.038923
Coenzyme A	−0.36718	−2.9008	0.005374	0.038923
Kyn	0.36418	2.8735	0.005794	0.040726
Α-ketoglutarate	0.35619	2.8012	0.007053	0.048159

Compounds in italics correlated at FDR p < 0.05 with SLE, BILAG, and FAS scores

### Kynurenine accumulation is most prominently reversed by NAC treatment in vivo

As previously shown, GSH is depleted in lupus PBL (Gergely et al. 2002) and its reversal by treatment with NAC was safe and effective in reducing disease activity in patients with SLE (Lai et al. 2012). Therefore, we investigated the metabolomic impact of NAC relative to placebo by performing a two-factor (NAC versus placebo) time series analysis within individual subjects using samples acquired during the randomized double-blind clinical trial (Lai et al. 2012). Two-way within-subject ANOVA was performed and the interaction of drug with time was analyzed by comparing data acquired after treatment for 1 month (visit 2), 2 months (visit 3), and 3 months (visit 4) relative to baseline (visit 1). Following Bonferroni’s correction for 197 detected metabolites, NAC treatment significantly reduced Kyn levels relative to placebo (raw p = 2.8 × 10^−7^, FDR corrected p = 6.6 × 10^−5^). 10 additional metabolites, were affected by NAC with raw p < 0.05 (Table 5), including mTOR-regulated orotate (Ben-Sahra et al. 2013). A multivariate extension of ANOVA, ASCA identified nine metabolites, also including Kyn as the top compound that best modeled the interaction between time and drug (Table 6).

**Table 5:** Effect of NAC relative to placebo by two-way ANOVA using time series (relative to visit 1) with drug interaction (NAC versus Placebo)

FDR p	Time	Drug	Interaction
*Kyn*	0.66378	0.85943	*2.88E−07*
6-Phospho-d-gluconate	0.48715	0.39983	0.001036
Purine	0.00876	0.20384	0.010943
Coenzyme A	0.23545	0.57885	0.011679
Pyroglutamic acid	0.22904	0.48637	0.014384
Orotate	0.20237	0.11511	0.021869
Betaine aldehyde	0.41832	0.73716	0.027097
Glucosamine	0.2869	0.54004	0.031178
5-Phosphoribosyl-1-pyrophosphate	0.11376	0.83987	0.034412
*N*-Acetyl-glucosamine	0.89918	0.55091	0.034934
2-Deoxyglucose-6-phosphate	0.46704	0.68485	0.038873
Flavone	0.46578	0.44401	0.049396

Only Kyn (in italics) survived Bonferroni’s correction for 197 detected metabolites

**Table 6:** Effect of NAC relative to placebo on metabolome of lupus PBL using ASCA

Compound	Leverage	SPE
Kyn	0.083705	11.42296
Purine	0.069117	2.185625
Coenzyme A	0.067726	3.532872
dUTP	0.060871	0.666888
Shikimate 3-phosphate	0.055991	4.9343
*N*-acetyl-glucosamine	0.053794	5.661865
Flavone	0.049883	10.8032
Ethanolamine	0.04957	4.714459
5-Methyl-THF	0.044736	5.661408

The significant variables have been identified based on the leverage and the Squared Prediction Errors (SPE) associated with each variable. Variables with low SPE and higher leverage modeled well after the major patterns: time, drug, and interaction of time and drug. The table shows metabolites that modeled well by interaction effect between Time and Drug

**Fig. 4:**
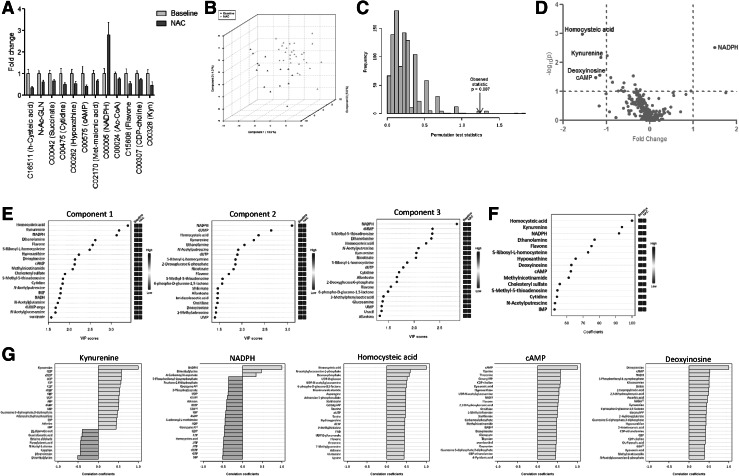
Effect of in vivo NAC treatment on metabolite concentrations in lupus PBL by comparing pre-treatment samples obtained at visit 1 to samples obtained at visits 2–4 during biologically and clinical effective administration of NAC at doses of 2.4 g/day and 4.8 g/day. **a** Metabolite concentrations affected by NAC at p < 0.05 on the basis of ANOVA. **b** 3-dimensional score plot of PLS-DA with components 1, 2, and 3, accounting for 18.9, 5.8, and 4.3 % of the total variance. **c** Validation of PLS-DA by permutation test p = 0.007. **d** Volcano plot is a combination of fold change (log2 FC: X axis) and *t* test p values (−log10 p: Y axis). **e** Variable importance in projection (VIP) scores of 15 top contributors to the PLS-DA components 1–3. **f** Coefficient-based importance measures of the top 15 contributors to components 1–3. **g** Correlation plots showing the compounds as *horizontal bars*, with colors in *light pink* indicating positive correlations and *light blue* indicating negative correlations with top lupus-associated metabolites identified in the volcano plot (**d**). Values reflect Pearson’s correlation coefficients between metabolite concentrations at FDR p < 0.05 calculated by MetaboAnalyst (Color figure online)

**Fig. 5:**
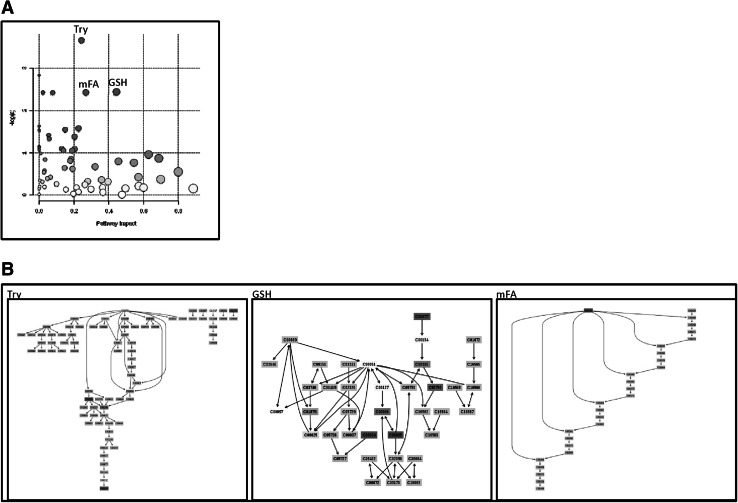
Effect of NAC on the metabolome of lupus PBL. **a** Global metabolome and pathway analyses were performed between 15 pre-treatment samples obtained at visit 1 and 29 samples obtained at visits 2–4 during biologically and clinical effective administration of NAC at doses of 2.4 g/day in 17 patients and 4.8 g/day in 12 patients. **b** Effect of NAC on metabolic pathways. 3 pathways were affected by NAC treatment, tryptophan, GSH, and mitochondrial fatty acid elongation (mFA); none of these pathway effects survived FDR or Bonferroni correction

The impact of NAC on the metabolome of lupus PBL was further evaluated by pathway analysis comparing pre-treatment samples obtained at visit 1 to samples obtained at visits 2–4 during biologically and clinical effective administration of NAC at doses of 2.4 and 4.8 g/day. In contrast to the robust differences between the global metabolomes of lupus and control PBL at baseline, NAC treatment affected a narrow range of metabolites (Fig. 4a) without global effect on the metabolome (Fig. 5a). As shown in Fig. 4a, NAC treatment markedly increased NADPH (+281 ± 57 %, p = 0.035),while it diminished Kyn (−55 ± 16 %, p = 0.04), HCA (−66 ± 7 %, p = 0.0008), N-acetyl-glutamine (−37 ± 7 %, p = 0.015), succinic acid (−34 ± 6 %, p = 0.025), cytidine (−50 ± 9 %, p = 0.027), hypoxanthine (−45 ± 10 %, p = 0.032), cAMP (−58 ± 6 %, p = 0.033), methyl-malonic acid (−33 ± 7 %, p = 0.035), acetyl-coenzyme A (−27 ± 4 %, p = 0.044), flavone (−46 ± 11 %, p = 0.045), and CDP-choline (−28 ± 7 %, p = 0.048). At 95 % confidence interval (CI), the effects of NAC treatment on Kyn, HCA, and NADPH were also significant (data not shown). Pathway analysis only showed an influence on tryptophan metabolism, mainly through Kyn, xanthurenic acid, and Ac-CoA, and lesser effects on GSH metabolism and fatty acid elongation in mitochondria; none of these pathway effects survived FDR correction (Fig. 5b).

**Fig. 6:**
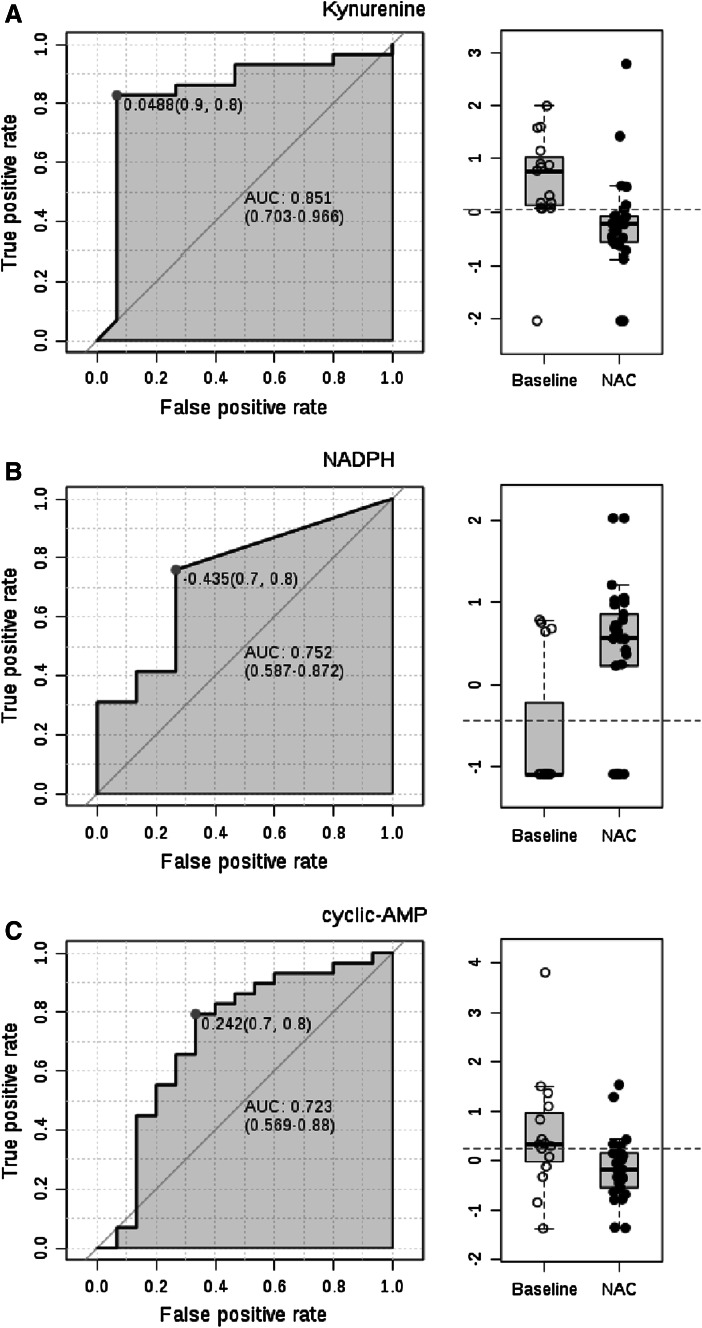
Receiver operating characteristic (ROC) curve logistic regression analysis of the metabolomic effects by NAC in lupus PBL. For each of the top three metabolites, the *left panel* shows the area under the ROC curve (AUC), true positive and false positive rates, and confidence interval (CI), the *right panel* shows the concentrations of metabolites in PBL before (baseline) and during NAC treatment (NAC). AUC logistic regression analysis indicated that Kyn (AUC = 0.851), NADPH (AUC = 0.752), and cAMP had the greatest specificity and sensitivity to detect the metabolomic effects of NAC in SLE (AUC = 0.723)

PLS-DA of metabolite concentrations effectively discriminated between baseline and NAC-treated lupus PBL (Fig. 3b), as validated by permutation test p = 0.007 (Fig. 4c).NAC decreased Kyn, HCA, deoxyinosine, and cAMP and increased NADPH, as shown by Volcano plot (Fig. 4d). NADPH, HCA, and Kyn had the greatest PLS-DA VIP (Fig. 4e) and coefficient scores towards distinguishing lupus PBL obtained before treatment from lupus PBL obtained during NAC treatment (Fig. 4f). Pearson’s correlation analysis with top PLS-DA coefficients revealed significant interconnectedness among pathways represented by lupus-associated metabolites (Fig. 4g). Thus, NADPH negatively correlated with sedoheptulose 1,7-bisphosphate (SBP, r = −0.51; FDR p = 0.033) and HCA (r = −0.49; FDR p = 0.035). Kyn significantly correlated with 22 metabolites at FDR <0.05, dominated by purine nucleotides such as GDP (r = 0.58; FDR p = 0.003). HCA positively correlated with 49 metabolites at FDR p < 0.05, including sedoheptulose 7-phosphate (S7P; r = 0.43; p = 0.028), inositol (r = 0.41; p = 0.035), carbamoyl phosphate (r = 0.40; p = 0.036). cAMP correlated with taurine (r = 0.58; FDR p = 0.003) and 48 other metabolites, including GPP (r = 0.57; FDR p = 0.003), CDP-choline (r = 0.57; FDR p = 0.003), kynurenic acid (r = 0.56; FDR p = 0.003), 1-methyl-adenosine (r = 0.49; FDR p = 0.012), carbamoyl phosphate (r = 0.48; FDR p = 0.016), deoxyinosine (r = 0.47; FDR p = 0.016), inositol (r = 0.47; FDR p = 0.016), dihydroorotate (r = 0.45; FDR p = 0.019), and NADP (r = 0.43; FDR p = 0.025). Deoxyinosine correlated with cAMP (r = 0.52; FDR p = 0.021), NADH (r = 0.52; FDR p = 0.021), and Kyn (r = 0.45; FDR p = 0.052). These latter findings are consistent with the involvement of Kyn in nucleotide metabolism (Pileni et al. 1977). AUC logistic regression analysis indicated that Kyn (Fig. 6a), NADPH (Fig. 6b), and cAMP had the greatest specificity and sensitivity to detect the metabolomic effects of NAC in SLE (Fig. 6c).

### Kynurenine stimulates mTOR activity in DN T cells

**Fig. 7:**
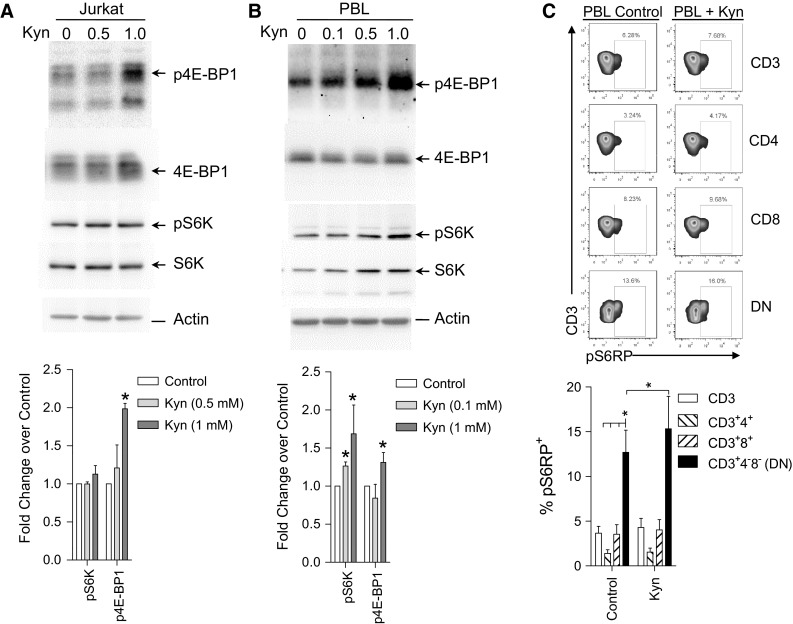
Activation of mTOR by kynurenine (Kyn). **a** Jurkat human T cells were incubated with or without Kyn at the indicated concentrations of 0.5 and 1 mM for 24 h and analyzed by western blot. mTORC1 activity was assessed by the levels of phosphorylated substrates, p4E-BP1 and pS6K, relative to actin control. **b** Western blot analysis of PBL from 8 healthy subjects incubated with or without Kyn at the indicated concentrations of 0.1, 0.5, and 1 mM for 24 h. C, Flow cytometry of intracellular pS6RP levels in CD4, CD8, DN T-cell subsets of 8 healthy subjects. While *top panels* show representative western blots (**a**, **b**) and flow cytometry dot plots (**c**), *bottom panels* indicate cumulative analyses. *reflect p values <0.05

Treatment of SLE patients with NAC resulted in the reversal of mTOR activation in T cells, most prominently in CD4^−^CD8^−^ double-negative (DN) T cells (Lai et al. 2012). These DN T cells have been implicated in elevated production of IL-4 (Chan et al. 2006) and IL-17 (Kato and Perl 2014) and stimulating anti-DNA production (Shivakumar et al. 1989) and organ damage (Crispin et al. 2008). Therefore, we examined whether the metabolites that were found to be accumulated in lupus PBL and regulated by treatment with NAC (Kyn, HCA, and cAMP), were actually capable of activating mTOR. Among these metabolites, only kynurenine activated mTOR complex 1 activity, as measured by phosphorylation of 4E-BP1, both in the Jurkat human T cell line and primary PBL (Fig. 7a, b). The effect of these metabolites on mTORC1 activity within T-cell subsets was measured via phosphorylation S6RP (Lai et al. 2012). T-cell subsets were analyzed by staining with antibodies to CD4, CD8, CD25, and FoxP3. In accordance with previous findings (Lai et al. 2012), mTOR was most elevated in DN T cells relative to CD3^+^, CD4^+^, or CD8^+^ T cells (p < 0.001; Fig. 7c) and CD4^+^CD25^+^FoxP3^+^ Tregs (p < 0.001; data not shown). Kyn stimulated mTOR in DN T cells (Fig. 7c). As a control amino acid of kynurenine metabolism, tryptophan (0.1, 1, and 10), which was neither accumulated in SLE nor influenced by NAC, did not stimulate mTOR activity (data not shown). Dibutyryl cAMP (db-cAMP), a cell-permeable derivative of cAMP, reduced mTOR activity in all T-cell subsets (data not shown). Inhibition of mTOR by cAMP was consistent with earlier findings (Xie et al. 2011).

## Discussion

**Fig. 8:**
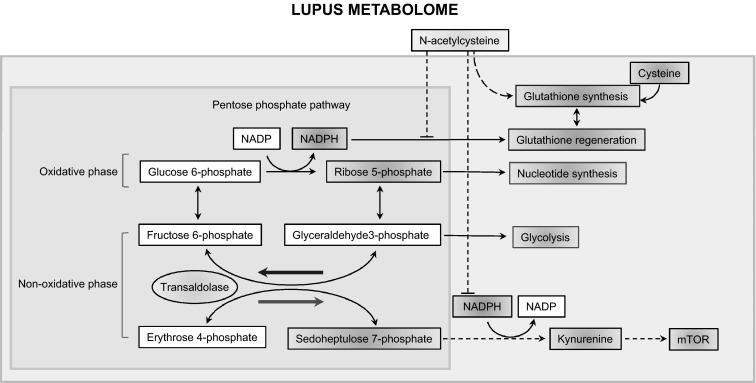
Schematic diagram of the prominent metabolomic changes that impact the pentose phosphate pathway (PPP) in patients with SLE. *Red* and *blue* arrows mark the forward and reverse reactions in the PPP, which are catalyzed by transaldolase (TAL), respectively (Perl et al. 2011). Metabolites are highlighted in *red* or *blue*, which reflects their increase or decrease in lupus PBL. The PPP is connected with the depletion of cysteine and the accumulation of homocysteic acid (HCA) and kynurenine (Kyn). Therapeutic intervention with *N*-acetylcysteine (NAC) reverses the accumulation of Kyn and the activation of mTOR, which are thus considered redox-sensitive drivers of lupus pathogenesis (Color figure online)

The present study reveals robust metabolome changes in SLE with a most prominent impact on the PPP. This pathway supplies essential metabolites, R5P for nucleotide biosynthesis and cell proliferation, and NADPH for antioxidant defenses. The accumulation of S7P also implicates the PPP in SLE, given that this metabolite is unique to this pathway (Perl et al. 2011). S7P is a substrate of transaldolase (TAL), and it is known to only accumulate in the deficiency of this enzyme in mice (Perl et al. 2011; Hanczko et al. 2009) and humans (Qian et al. 2008). The PPP is tightly linked to cysteine metabolism and GSH homeostasis by providing NADPH, which is essential for the regeneration of GSH from its oxidized form, GSSG (Perl 2013) (Fig. 8). As shown in this study, cysteine, which is the rate-limiting factor of de novo GSH synthesis (Lu 2013), was profoundly diminished in lupus PBL. Importantly, the loss of cysteine was associated with the accumulation of its oxidized product, cystine, as well as Met-SO (Haenold et al. 2008) and HCA (Boldyrev 2009; Go and Jones 2011), all of which represent metabolic evidence of oxidative stress in SLE. While the depletion of cysteine and the accumulation of cystine and Met-SO clearly indicate increased oxidative stress in SLE, none of these compounds or the unique PPP substrates R5P or S7P correlated with disease activity of untreated patients. In contrast, metabolites connecting multiple pathways, such as DHAP, common to both the PPP and glycolysis, and OAA, common to the both TCA and glycolysis, correlated with SLEDAI, BILAG, and FAS scores, suggesting that increased flux among these pathways are involved in disease activity.

**Fig. 9:**
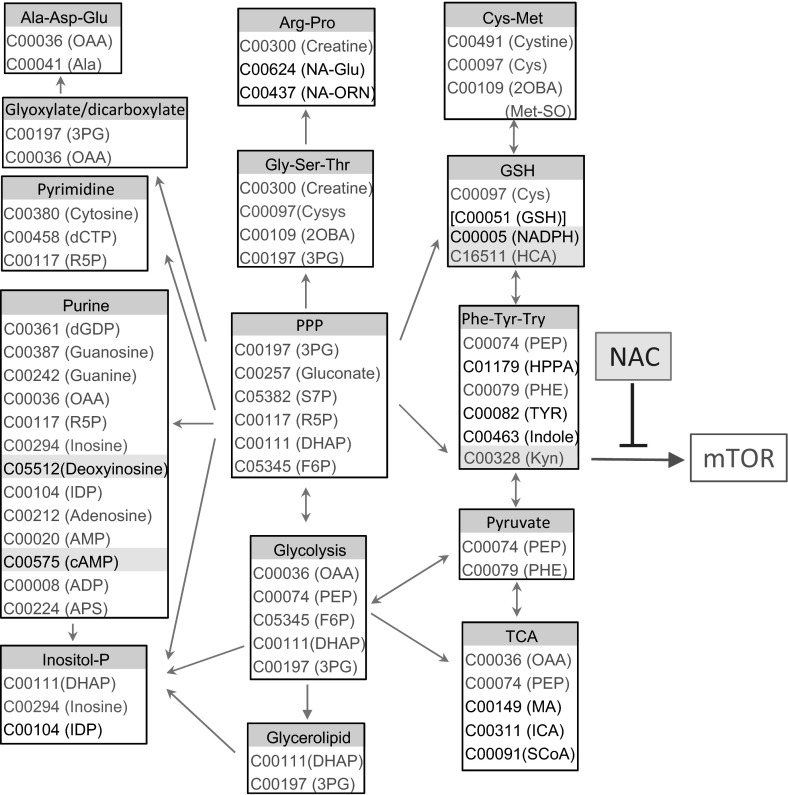
Schematic interaction of metabolomic changes in lupus PBL via the PPP. Altered compounds are identified by standard KEGG codes and acronyms in parentheses: compounds in red indicate metabolites with increased levels in lupus PBL; compounds in blue indicate metabolites with reduced levels in lupus PBL; compounds affected by NAC treatment are highlighted in *yellow*; compounds in *black* indicate metabolites with increased levels in lupus PBL but not connected to the PPP. *Green arrows* indicate pathways connected by common metabolites, with *arrowheads* marking directionality of metabolic flux. *Red arrows mark* metabolites capable of directly activating mTOR (Color figure online)

As shown in this study, the prominent and NAC-responsive accumulation of Kyn and its ability to activate mTOR in DN T cells constitute a metabolic pathway of T cell activation and lineage development in general. This mechanism is particularly significant for lupus pathogenesis, given that DN T cells are a primary source of pro-inflammatory IL-4, IL-17 and necrotic debris (Lai et al. 2013). The accumulation of Kyn may be a result of decreased catabolism by Kyn hydroxylase owing to NADPH dependence of this enzyme (Breton et al. 2000). Along these lines, NAC dramatically augmented the levels of NADPH, which can occur through sparing of NADPH via the enhancement of de novo GSH synthesis by NAC (Perl et al. 2011; Hanczko et al. 2009). Thus, the marked suppression of Kyn in NAC-treated patients can be attributed to the NADPH sparing effect of NAC (Hanczko et al. 2009). The reversal of HCA accumulation may represent a direct effect of NAC on cysteine metabolism. By contrast, NAC treatment did not reduce elevated levels of unique PPP sugars, S7P and R5P, suggesting that altered PPP activity lies upstream of GSH depletion in the order of metabolic signaling defects in SLE. NAC also affected other metabolic pathways that are centrally connected to the PPP (Fig. 9). mTOR complex 1 (mTORC1), which was measured here via phosphorylation of its downstream substrate, pS6RP, is stimulated by oxidative stress (Sarbassov and Sabatini 2005) and the lysosomal accumulation of amino acids (Bar-Peled et al. 2013). However, the identity of specific amino acids that are sensed by mTORC1 are presently unknown (Jewell et al. 2013). Kynurenine has been recently linked to oxidative damage in the eye (Linetsky et al. 2014). Therefore, the NAC-responsive accumulation of Kyn may be particularly relevant for understanding the fundamental biology of mTORC1 activation by amino acids (Jewell et al. 2013) under oxidative stress (Sarbassov and Sabatini 2005). Tryptophan breakdown products, including Kyn, have been found to possess both antioxidant (Gostner et al. 2015; Grewal et al. 2014) and pro-oxidant properties (Linetsky et al. 2014). Therefore, further studies are clearly warranted to address the role of Kyn in oxidative stress of patients with SLE.

In summary, this study documents profound changes in the metabolome of lupus PBL with a dominant impact on the PPP that reflects greater demand for nucleotides and oxidative stress. The hereby discovered accumulation of Kyn, which is metabolically linked to increased PPP activity and responds to treatment with NAC, is identified as potential contributor to mTOR activation in SLE.

## Electronic supplementary material

Below is the link to the electronic supplementary material.

Supplementary material 1 (PDF 1309 kb)

## Abbreviations

2OBA: 2-Oxobutanoate
3PG: 3-Phosphoglycerate
AUC: Area under the receiver operating characteristic (ROC) curve
BILAG: British Isles Lupus Assessment Group
cAMP: Cyclic AMP
db-cAMP: Dibutyryl cAMP
DHAP: Dihydroxy-acetone-phosphate
DN T: CD3^+^CD4^−^CD8^−^ double-negative T cell
E4P: Erythrose 4-phosphate
FAS: Fatigue assessment scale
FDR: False discovery rate
G1P: Glucose 1-phosphate
G6P: Glucose 6-phosphate
GA3P: Glyceraldehyde 3-phosphate
GL6P: Gluconolactone 6-phosphate
GPP: Geranyl pyrophosphate
GSH: Reduced glutathione
GSSG: Oxidized glutathione
HCA: Homocysteic acid
HCY: Homocysteine
HE: Hydroethidine;ROI sensor
IAA: Indol-3-acetic acid
Kyn: Kynurenine
LC–MS/MS: Liquid chromatography-tandem mass spectroscopy
Met-SO: Methionine sulfoxide
mTOR: Mammalian/mechanistic target of rapamycin
NAC: *N*-acetylcysteine
O8P: Octulose 8-phosphate
OAA: Oxaloacetate
PBL: Peripheral blood lymphocytes
PBS: Phosphate buffered saline
PCA: Principal component analysis
PLS-DA: Partial least square-discriminant analysis
PI: Propidium iodide
R5P: Ribose 5-phosphate
RFI: Relative fluorescence intensity
ROC: Receiver operating characteristic
S7P: Sedoheptulose 7-phosphate
SBP: Sedoheptulose 1,7-bisphosphate
SLE: Systemic lupus erythematosus
SLEDAI: Systemic lupus erythematosus disease activity index
SPE: Squared prediction errors
T6P: Trehalose 6-phosphate
VIP: Variable importance in projection
