# Supplementary material for: Comprehensive metabolome analyses reveal N-acetylcysteine-responsive accumulation of kynurenine in systemic lupus erythematosus: implications for activation of the mechanistic target of rapamycin
Source: Metabolomics. 2015 Jan 20;11(5):1157–74. doi: 10.1007/s11306-015-0772-0 (PMC4559110; doi:10.1007/s11306-015-0772-0)
Supplement: Supplementary file 1 — Supplementary material 1 (PDF 1309 kb) [file 11306_2015_772_MOESM1_ESM.pdf]

SUPPLEMENTAL FIGURE S1

FIGURE S1 IS TO BE VIEWED WITH HIGH RESOLUTION DIGITAL ZOOMING

Figure S1. Pathway view of quantitative changes in compound concentrations in PBL samples of 36 SLE patients relative to PBL samples of 42 healthy subjects matched for age within 10 years, gender, and ethnic background. 26 pathways with false discovery rate (FDR) p values < 0.05 are arranged according to the magnitude of impact, which is based on the number of detected metabolites with significant p value and their node of importance. KEGG codes represent individual metabolites that are colored according to their absence (blue) or presence (yellow, orange, or red with the relative decrease of Holm p value).

Fig. S1

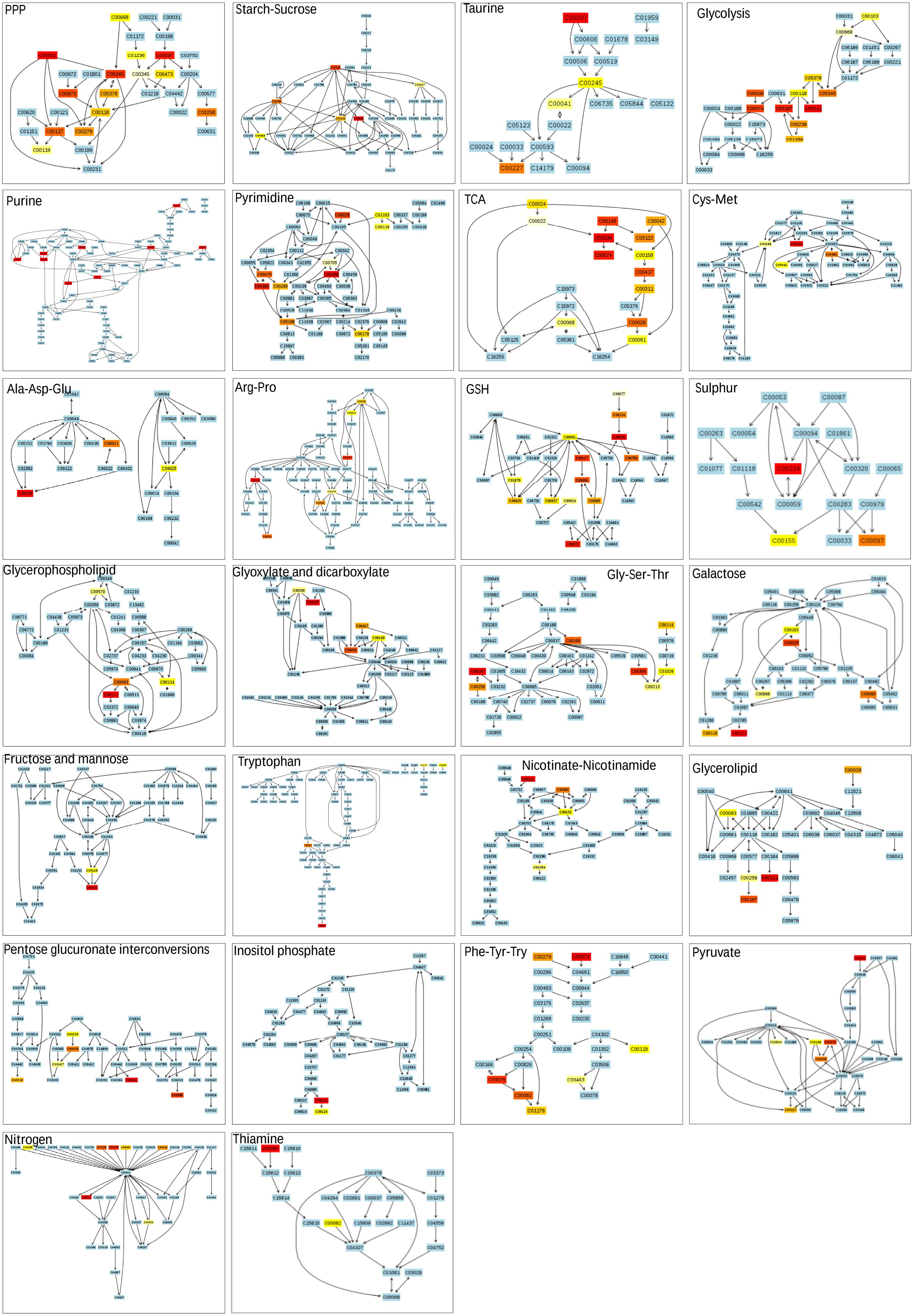
PPP
Starch-Sucrose
Taurine
Glycolysis
Purine
Pyrimidine
TCA
Cys-Met
Ala-Asp-Glu
Arg-Pro
GSH
Sulphur
Glycerophospholipid
Glyoxylate and dicarboxylate
Gly-Ser-Thr
Galactose
Fructose and mannose
Tryptophan
Nicotinate-Nicotinamide
Glycerolipid
Pentose glucuronate interconversions
Inositol phosphate
Phe-Tyr-Try
Pyruvate
Nitrogen
Thiamine
